# Histopathological findings, phenotyping of inflammatory cells, and expression of markers of nitritative injury in joint tissue samples from calves after vaccination and intraarticular challenge with *Mycoplasma bovis* strain 1067

**DOI:** 10.1186/s13028-014-0045-3

**Published:** 2014-08-19

**Authors:** Vemuri Rama Devi, François Poumarat, Dominique Le Grand, Renate Rosengarten, Kathrin Hermeyer, Marion Hewicker-Trautwein

**Affiliations:** 1 Department of Pathology, University of Veterinary Medicine Hannover, Bünteweg 17, Hannover, D-30559, Germany; 2 Anses, Lyon Laboratory, UMR Mycoplasmoses des Ruminants, 31 Avenue Tony Garnier, Lyon, F-69364 cedex 07, France; 3 Université de Lyon, VetAgro Sup, UMR Mycoplasmoses des Ruminants, 1 Avenue Bourgelat, Marcy-L’Étoile, F-69280, France; 4 Institute of Bacteriology, Mycology and Hygiene, University of Veterinary Medicine Vienna, Veterinärplatz 1, Vienna, A-1210, Austria; 5 Current address: Department of Veterinary Pathology, NTR College of Veterinary Science, Sri Venkateswara Veterinary University, Gannavaram 521 102, India; 6 Current address: Vet Med Labor GmbH, Division of IDEXX Laboratories, Mörikestrasse 28/3, Ludwigsburg, D-71636, Germany

**Keywords:** Mycoplasma bovis, Arthritis, Vaccination, Variable surface protein antigens, MHC class II, Inducible nitric oxide, Nitrotyrosine

## Abstract

**Background:**

The pathogenesis of caseonecrotic lesions developing in lungs and joints of calves infected with *Mycoplasma bovis* is not clear and attempts to prevent *M. bovis*-induced disease by vaccines have been largely unsuccessful. In this investigation, joint samples from 4 calves, i.e. 2 vaccinated and 2 non-vaccinated, of a vaccination experiment with intraarticular challenge were examined. The aim was to characterize the histopathological findings, the phenotypes of inflammatory cells, the expression of class II major histocompatibility complex (MHC class II) molecules, and the expression of markers for nitritative stress, i.e. inducible nitric oxide synthase (iNOS) and nitrotyrosine (NT), in synovial membrane samples from these calves. Furthermore, the samples were examined for *M. bovis* antigens including variable surface protein (Vsp) antigens and *M. bovis* organisms by cultivation techniques.

**Results:**

The inoculated joints of all 4 calves had caseonecrotic and inflammatory lesions. Necrotic foci were demarcated by phagocytic cells, i.e. macrophages and neutrophilic granulocytes, and by T and B lymphocytes. The presence of *M. bovis* antigens in necrotic tissue lesions was associated with expression of iNOS and NT by macrophages. Only single macrophages demarcating the necrotic foci were positive for MHC class II. Microbiological results revealed that *M. bovis* had spread to approximately 27% of the non-inoculated joints. Differences in extent or severity between the lesions in samples from vaccinated and non-vaccinated animals were not seen.

**Conclusions:**

The results suggest that nitritative injury, as in pneumonic lung tissue of *M. bovis*-infected calves, is involved in the development of caseonecrotic joint lesions. Only single macrophages were positive for MHC class II indicating down-regulation of antigen-presenting mechanisms possibly caused by local production of iNOS and NO by infiltrating macrophages.

## Background

Caseonecrotic pneumonia in calves and cattle is considered a distinctive lesion caused by *Mycoplasma bovis*[1]. In animals with pneumonia caused by *M. bovis* arthritis frequently develops secondary to respiratory infection [2]. Within the joint tissue of *M. bovis* infected animals, as in lungs, necrotic alterations occur [2]–[5]. The pathogenesis of necrotizing joint lesions is not clear. Recent findings in necrotic lung foci suggest that the production of nitric oxide (NO) and peroxynitrite by inducible nitric oxide (iNOS)- and nitrotyrosine (NT)-expressing macrophages is potentially involved in the development of these tissue lesions [6]–[8]. Furthermore, findings in necrotic lung tissue of such calves indicate that surface protein antigen variation of *M. bovis* occurs *in vivo* and that local antigen-presenting mechanisms are possibly down-regulated due to the production of iNOS and NO [6],[7],[9].

Several efforts have been undertaken to develop vaccines to prevent *M. bovis* induced disease. Certain vaccines give partial protection from respiratory disease and reduce the spread of *M. bovis* to internal organs including the joints but other attempts have largely been unsuccessful [10]. A deeper knowledge of the morphological changes due to *M. bovis* in joint tissue may help to understand better the mechanisms of the disease and may be a basis for future interventions such as development of drugs or improved vaccines.

In the present investigation, joint tissue samples from 2 vaccinated and 2 non-vaccinated calves of a vaccination experiment were examined by applying histological and immunohistochemical techniques. One aim of this study was to characterize the histopathological findings and the types of inflammatory cells, the MHC class II expression, and the expression of markers for nitritative stress, i.e. iNOS and NT in samples from the inoculated joint and several non-inoculated joints of the 4 calves. A second aim was to examine the joint samples both for the presence of *M. bovis* antigens including variable surface protein (Vsp) antigens and for *M. bovis* organisms.

## Methods

### Calves and inoculation

In this study, 48 synovial membrane samples from 4 *M. bovis*-inoculated calves were examined. The calves had been injected intraarticularly in an inoculation study described elsewhere, which was performed to evaluate the potential of a formalinized vaccine for prevention of *M. bovis* arthritis [11] by following a previously used infection protocol [12]. The animal experiment was conducted during the years 1991–1992 in a registered facility of the governmental institution Anses, UMR Mycoplasmoses des Ruminants, Lyon Laboratory, Lyon, France. The animals were kept in registered holding rooms for cattle and all stages of the experimental protocol were performed and supervised by certified veterinarians. These experiments were carried out by veterinarians under the authority of the French government. Briefly, the experiment was carried out with 22 conventionally reared calves, aged between 8 and 15 days. All calves were examined for the presence of antibodies to *M. bovis* by an indirect haemagglutination test [13]. None of the animals had serological evidence of previous exposure to *M. bovis* as proved by 4 successive negative blood samplings at 8 days intervals before the beginning of the experiment. At the age of 4 to 5 weeks, eleven calves were vaccinated intramuscularly with 5 × 10^10^ formalin-inactivated *M. bovis* organisms with aluminium hydroxyde and Quail A saponine as adjuvants (50:50) and 4 weeks later, a second vaccination with the same dose of the same vaccine was performed. For production of the vaccine and for challenging the animals *M. bovis* strain 1067, which had been isolated from a cow with mastitis [14], was used. Three weeks after the second vaccination, the 11 vaccinated calves and 10 non-vaccinated calves were challenged by inoculating 0.5 ml of culture medium containing 2.5 × 10^7^ viable organisms of *M. bovis* into the joint space of the right carpal joint. At challenge, the 21 calves were 11 to 12 weeks old. Another calf (No. 5), which served as control, was inoculated into the joint space of the right carpal joint with the same volume of sterile *Mycoplasma* culture medium. Before intraarticular inoculation, sedation and analgesia were performed by intramuscular injection of 0.1 mg/kg xylazine (Rompun, Bayer Santé-Division Santé Animale). Serum samples from all animals were screened weekly throughout the experiment for antibodies to *M. bovis* by using a passive haemagglutination test [13].

### Necropsy and sampling

One vaccinated and one non-vaccinated calf were euthanized daily by injection of embutramide, mebezonium iodide, and tetracaine hydrochloride (T61®, MSD Santé Animale) and necropsied from day 10 after intraarticular inoculation. Euthanasia and necropsy of the control calf were carried out 13 days after intraarticular injection of sterile culture medium. Synovial membrane samples were collected from both fore limbs (elbow, carpal, and metacarpo-phalangeal joints) and from both hind limbs (femoro-tibial, tarsal, and metatarso-phalangeal joints). The samples were analyzed for the presence of mycoplasmas by standard cultivation procedures. Additional samples from all joints were fixed in 10% neutral buffered formalin and embedded in paraffin wax, sectioned (4 μm) and stained with haematoxylin and eosin (H&E). In the present investigation, samples from 2 vaccinated calves and from 2 non-vaccinated calves from the vaccination experiment with subsequent intra-articular challenge were randomly selected. They had been necropsied between days 10 and 14 after intraarticular inoculation with *M. bovis* (Tables 1 and 2). From each of the 4 animals, 12 joint samples were examined. Furthermore, 12 samples from the same joints of the control calf were examined.

**Table 1:** **Microbiological findings in synovial tissue samples of 4 calves after intra-articular inoculation/challenge with** ***Mycoplasma bovis***

**Calf no.**	**Necropsy after inoculation/challenge (in days)**	**Right fore limb**			**Left fore limb**			**Right hind limb**			**Left hind limb**		
		**A1**^**a**^	**B1**^**b,c**^	**C1**^**d**^	**A1**	**B1**	**C1**	**A2**^**e**^	**B2**^**f**^	**C2**^**g**^	**A2**	**B2**	**C2**
1^h^	12	+	+	-	+	+	+	-	+	-	-	+	+
2^h^	14	-	+	-	-	-	-	-	-	-	-	-	-
3^i^	10	+	+	-	-	-	-	+	-	+	-	-	+
4^i^	12	-	+	-	-	+	-	-	-	-	-	-	-

^a^elbow joint. ^b^carpal joint. ^c^inoculated joint. ^d^metacarpo-phalangeal joint.

^e^femoro-tibial joint. ^f^tarsal joint. ^g^metatarso-phalangeal joint.

^h^non-vaccinated animal. ^i^ vaccinated animal.

+isolation of *M. bovis* positive.

-isolation of *M. bovis* negative.

**Table 2:** **Histopathological findings in 4 calves after intra-articular inoculation/challenge with** ***Mycoplasma bovis***

**Calf no.**	**Necropsy after inoculation/challenge (in days)**	**Right fore limb**			**Left fore limb**			**Right hind limb**			**Left hind limb**		
		**A1**^**a**^	**B1**^**b,c**^	**C1**^**d**^	**A1**	**B1**	**C1**	**A2**^**e**^	**B2**^**f**^	**C2**^**g**^	**A2**	**B2**	**C2**
1^h^	12	+^j^	N^k^,+++^l^	-^m^	-	++^n^	N,+++	+	++	-	-	N,+++	-
2^h^	14	-	N,+++	+	-	+	N,+	+	+	-	+	+	-
3^i^	10	-	N,+++	na^o^	-	na	na	++	na	+	-	na	-
4^i^	12	-	N,+++	na	na	-	na	na	+	na	na	-	na

^a^elbow joint. ^b^carpal joint. ^c^inoculated joint. ^d^metacarpo-phalangeal joint.

^e^femoro-tibial joint. ^f^tarsal joint. ^g^metatarso-phalangeal joint.

^h^non-vaccinated animal. ^i^vaccinated animal.

^j^mild inflammatory cell infiltration. ^k^necrosis. ^l^marked inflammatory cell infiltration.

^m^no abnormalities detected.

^n^moderate inflammatory cell infiltration.

^o^no sample available.

### Antibodies and immunohistochemistry

Paraffin sections were examined for *M. bovis* antigens, calprotectin (S100A8 and S100A9)-expressing macrophages, MHC class II antigen, iNOS and NT by applying the avidin-biotin-peroxidase (ABC) method and antibodies as shown in Table 3. Following dewaxing and blocking of endogenous peroxidase activity sections were pretreated for antigen retrieval (Table 3). All primary antibodies were incubated for approximately 16–18 h at 4°C. Thereafter, sections were incubated with biotin-conjugated antibodies to rabbit IgG or mouse IgG or IgM. For reactions with the monoclonal antibody (mAb) pool, a mixture (1:1) of secondary biotin-conjugated antibodies to mouse IgG and mouse IgM (each diluted 1:200) was used. After incubation with the ABC solution, the chromogen AEC was used for sections incubated with antibodies to iNOS and NT, for the other sections DAB was applied. Counterstaining was performed with Mayer’s haematoxylin. As positive controls for detecting *M. bovis* antigen lung sections from a calf from another experiment, which had been euthanised after respiratory infection with *M. bovis* strain 1067, were used. Sections of normal bovine lymph node tissue served as positive controls for immunolabelling of T and B lymphocytes, macrophages, and MHC class II antigen. As positive control for labelling of iNOS and NT, lung sections from normal cattle were used.

**Table 3:** Antibodies used in this study

**Antibody designation**	**Type or isotype**	**Specificity**	**Dilution**	**Antigen retrieval**	**References/source**
D490	Rabbit hyperimmune serum	*M. bovis*	1:1800	0.25% trypsin^a^ (37°C, 60 min)	[26]
mAb^b^ pool	Mouse IgG and IgM	*M. bovis*	1:2000	0.25% trypsin (37°C, 60 min)	Chemicon
1A1	Mouse IgG1	*M. bovis* VspA, C, and other Vsps^c^	1:200	0.25% trypsin (37°C, 60 min)	[27]
1E5	Mouse IgM	*M. bovis* VspA, B, C	undiluted	0.25% trypsin (37°C, 60 min)	[26],[28],[29]
Anti-CD3	Rabbit IgG	CD3^+^ T lymphocytes	1:300	Demasking solution G^d^ (95°C,	DakoCytomation
BAQ44A	Mouse IgM	B lymphocytes	1:100	20 min) 0.01 M citric buffer (microwave 95°C, 10 min)	VMRD; [23]
Anti-MRP8 (S100A8)	Mouse IgG	Human MRP8^e^	1:600	Pronase E^f^ (37°C, 20 min) [26],	BMA Biomedicals; [30]
Anti-MRP14 (S100A9)	Mouse IgG	Human MRP14^g^	1:8000	0.01 M citrate buffer (microwave 95°C, 10 min)	BMA Biomedicals; [30]
TAL.1B5	Mouse IgG1	α-chain of human leukocyte antigen (HLA-DR)	1:4000	0.01 M citrate buffer (microwave 95°C, 15 min)	DakoCytomation
Anti-iNOS/NOS II	Rabbit IgG	iNOS	1:50	Saponine^f^ (37°C, 20 min)	[31],[32]; Biomol GmbH
Anti-NT	Rabbit IgG	NT	1:100	Pronase E (37°C, 20 min)	Millipore GmbH

^a^Fluka, Buchs, Switzerland. ^b^monoclonal antibody. ^c^variable surface proteins belonging to the Vsp protein family of *M. bovis*.

^d^BIOLOGO, Kronshagen, Germany. ^e^Calgranulin A ^f^VWR TM International GmbH, Darmstadt, Germany. ^g^Calgranulin B.

## Results

### Clinical and microbiological findings

The findings for all 22 calves of the vaccination experiment were described previously [11]. Briefly, all 21 *M. bovis*-inoculated animals developed arthritis at the inoculation site and became lame and recumbent. Subsequently, most of the large joints became markedly swollen. From all inoculated calves, *M. bovis* was reisolated from the right carpal joint. Bacteria other than *M. bovis* were not isolated from any of the joints. In samples of the control calf neither *M. bovis* organisms nor other bacteria were detected by cultivation. Cultivation of joint samples revealed that *M. bovis* was present in joint tissue samples of 8 of the 10 non-vaccinated calves and in 5 of the 11 vaccinated calves. Vaccinated calves showed a significant increase of antibodies to *M. bovis* before challenge. The difference between the number of *M. bovis*-infected joints after challenge between the groups of vaccinated and non-vaccinated animals was not statistically significant (Fisher’s Exact Test). On necropsy, except from the joints, no gross lesions were found in the lungs or any other organs.

### Microbiological, histopathological and immunohistochemical findings in synovial tissue samples

From a total of 44 non-inoculated joints from 2 non-vaccinated calves (Nos. 1 and 2) and 2 vaccinated calves (Nos. 3 and 4), *M. bovis* was isolated from 12 of them (27.3%) (Table 1). In 7 samples of 12 of these non-inoculated, *M. bovis*-positive joints, histopathological changes were seen while the samples from the other 5 *M. bovis*-positive joints were histologically unremarkable. From samples of 9 non-inoculated joints of the 4 calves with histopathological changes no *M. bovis* organisms or other bacteria were isolated (Tables 1 and 2).

**Figure 1:**
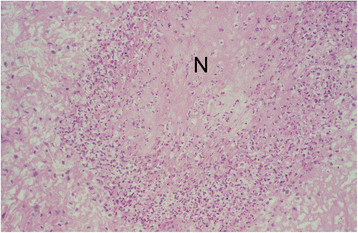
**Caseonecrotic lesion in the subsynovial connective tissue.** The necrotic centre (N) is demarcated and infiltrated by numerous neutrophilic granulocytes and macrophages. Left metacarpo-phalangeal joint of calf No. 1. H&E. ×200.

The subsynovial connective tissue of the inoculated right carpal joint of all 4 calves had multifocal, variable sized, irregularly formed caseonecrotic foci (Table 2). Such lesions were also found in the subsynovial connective tissue of 2 additional joints of calf No. 1 and in an additional joint of calf No. 2 (Table 2). Caseonecrotic foci were infiltrated with numerous morphologically intact and degenerate neutrophilic granulocytes (Figure 1). In the periphery of the foci many macrophages and fewer numbers of lymphocytes and plasma cells were found. There was multifocal slight hypertrophy and hyperplasia of synovial lining cells. Within the upper and deeper subsynovial tissue mainly perivascularly located accumulations of few lymphocytes and moderate numbers of plasma cells and macrophages were seen. Joint tissue samples from the control calf were histologically unremarkable.

**Figure 2:**
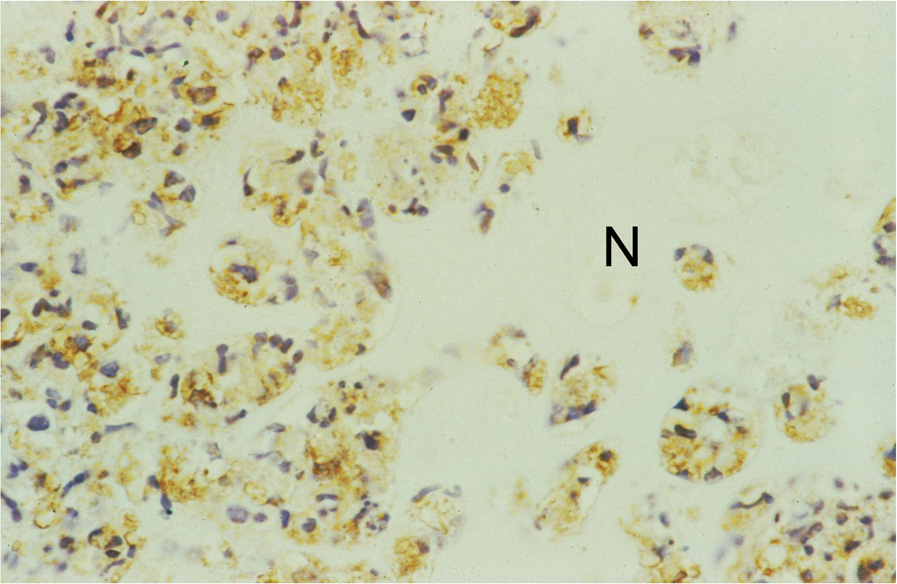
**Immunohistochemistry for*Mycoplasma bovis*antigen in an area of necrosis (N) with numerous macrophages with cytoplasmic antigen.** Right carpal joint of calf No. 2. Polyclonal antibody D490. ABC method. Chromogen DAB. ×400.

*M. bovis* antigens were exclusively found in necrotic tissue areas. *M. bovis* antigens were detected in the cytoplasm of macrophages and neutrophilic granulocytes demarcating the necrotic foci both with the polyclonal antibody D490 and with the mAb pool. Antigens were also found extracellularly within and especially at the periphery of necrotic tissue areas (Figure 2). With mAb 1A1 in all tissue sections with necrotic foci a similar labelling pattern was seen, although less antigen was present than in sections immunolabelled with antibody D490 and the mAb pool. With mAb 1E5 positive immunolabelling was only found in the necrotic lesions of the inoculated right carpus of calf No. 2.

**Figure 3:**
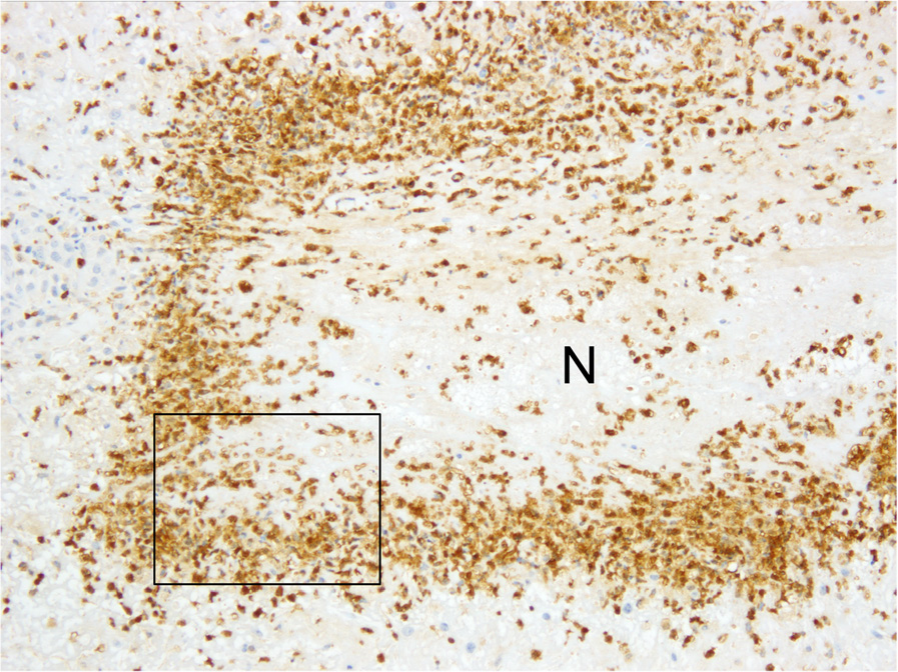
**Immunohistochemistry of a caseonecrotic lesion in the subsynovial connective tissue for S100A8 (MRP8).** The necrotic centre (N) is demarcated by numerous S100A8-expressing phagocytes. The frame identifies an enlarged area presented in Figure 4. ×100.

**Figure 4:**
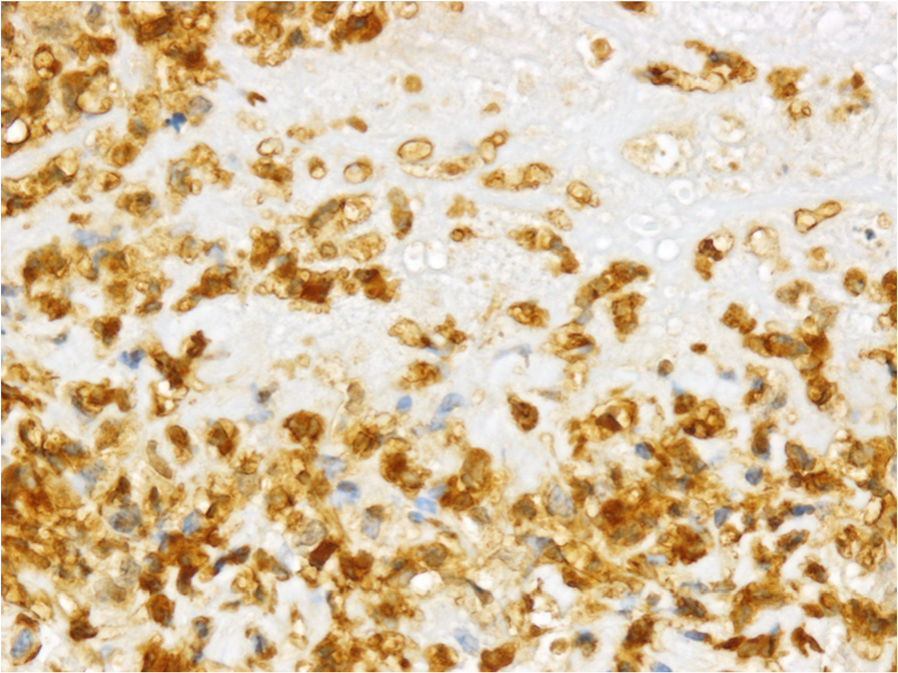
**A higher magnification of Figure**3**shows that the majority of macrophages have strong immunohistochemical labelling of the cytoplasm.** Left metacarpo-phalangeal joint of calf No. 1. ABC method. Chromogen DAB. ×400.

**Figure 5:**
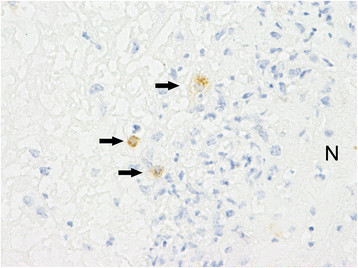
**Immunohistochemistry for MHC class II in a necrotic area in the subsynovial tissue.** There are only single MHC II-positive macrophages (arrows) surrounding the necrosis (N). Left metacarpal-phalangeal joint of calf No. 1. ABC method. Chromogen DAB. ×400.

**Figure 6:**
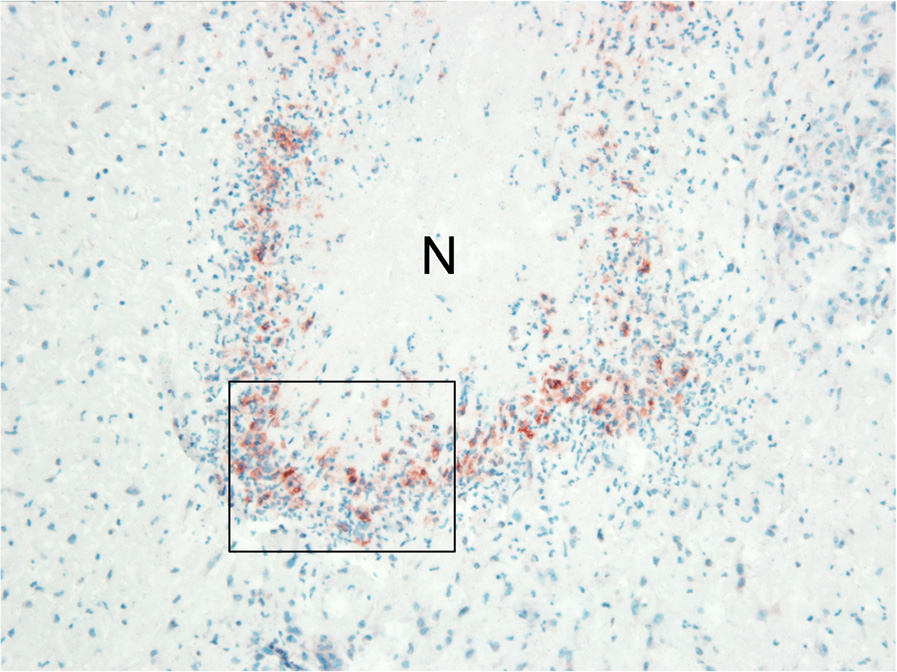
**Immunohistochemistry of a necrotic area (N) in the subsynovial tissue for iNOS.** The necrotic lesion is surrounded by numerous iNOS-positive phagocytes. The frame identifies an enlarged area presented in Figure 7. ×100.

**Figure 7:**
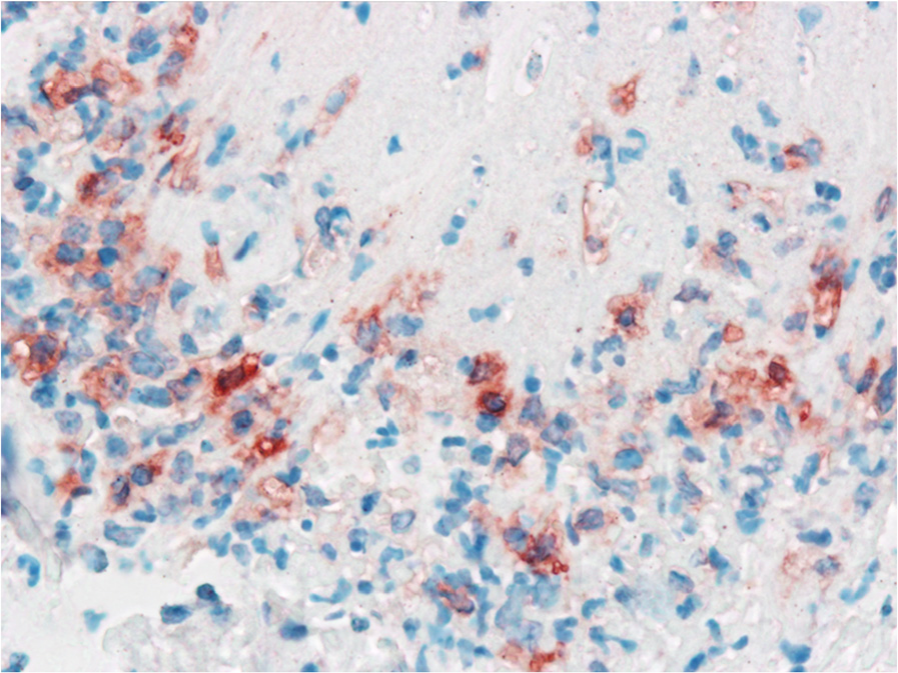
**A higher magnification of Figure**6**shows numerous macrophages with strong cytoplasmic immunolabelling.** Left metacarpal-phalangeal joint of calf No. 1. ABC method. Chromogen AEC. ×400.

Immunohistochemical phenotyping of inflammatory cells revealed that the majority of macrophages located within and especially at the periphery of caseonecrotic lesions were expressing S100A8 (Figures 3 and 4) and S100A9 (not shown). In the periphery of necrotic foci and around blood vessels within the deeper subsynovial tissue few T and B lymphocytes were present (not shown). MHC class II expression was found on approximately 10–20% of synovial lining cells and on many lymphocytes, macrophages and cells resembling dendritic cells located perivascularly within the non-necrotic connective tissue of synovial membranes (not shown). Macrophages in perinecrotic areas only occasionally were positive for MHC class II (Figure 5). Numerous macrophages demarcating the necrotic foci and also macrophages located perivascularly in the surrounding synovial tissue had cytoplasmic expression of iNOS (Figure 6 and 7) and NT. Such immunolabelling was occasionally seen in neutrophilic granulocytes in the periphery of necrotic lesions. Sections from the control calf were negative for *M. bovis* antigen and had approximately 10–20% MHC II-positive synovial lining cells. Furthermore, single T and B lymphocytes and single macrophages expressing both S100A8 and S100A9 were found in perivascular areas of sections from the control animal.

## Discussion

In this investigation, joint tissue samples from 4 calves, i.e. 2 vaccinated and 2 non-vaccinated, which were part of a vaccination experiment, were examined after intra-articular challenge with *M. bovis* for characterizing the histopathological findings including the types of inflammatory cells, MHC class II expression, the distribution of Vsp antigens of *M. bovis*, and the expression of markers for nitritative stress, i.e. iNOS and NT.

In all 4 calves, inoculation of *M. bovis* strain 1067 not only induced necrotizing and inflammatory lesions within their inoculated right carpal joint, but also resulted in necrotic and/or inflammatory lesions in several non-inoculated joints of the fore and/or hind limbs. The lesions within the synovial membranes were associated with the presence of the agent as demonstrated by immunohistochemistry and reisolation by cultivation. *M. bovis* antigens including Vsp antigens were still present at 10 and 14 days after infection and were exclusively found within caseonecrotic lesions, i.e. in the inoculated joints of all 4 calves and in 3 additional non-inoculated joints of 2 of the 4 calves.

The results of this investigation show that the necrotizing foci, as in lungs of infected calves [6],[7], are the main reservoir for *M. bovis* although they are demarcated and infiltrated by numerous neutrophilic granulocytes and macrophages. Furthermore, the presence of Vsp antigens both in the cytoplasm of macrophages and extracellularly as detected with the monoclonal antibodies 1A1 and 1E5 suggests that, as in lungs of *M. bovis*-infected calves [7], there is *in vivo* expression of *M. bovis* Vsp antigens. Variable expression of these proteins may be a major mechanism by which *M. bovis* evades the host immune response.

The vaccination experiment was performed to evaluate the potential effects of a formalinized vaccine for prevention of *M. bovis* arthritis. A protective effect of the vaccine preventing spreading of *M. bovis* from the inoculated joint to non-inoculated joints was not found. Cultural isolation revealed that *M. bovis* organisms, in addition to the 4 inoculated joints, were also present in approximately 27% of 44 non-inoculated joints. Statistically significant differences in the number of infected joints after challenge with *M. bovis* between vaccinated and non-vaccinated calf groups were not detected [11]. Furthermore, histopathology on the joint samples from the 4 calves performed in this study did not reveal any differences in extent or severity between the lesions in the 2 vaccinated and the 2 non-vaccinated animals.

Studies in *M. bovis*-infected calves with pneumonia suggest that they subsequently develop arthritis resulting from invasion across the airway or alveolar epithelium, or by lymphatic drainage from alveoli through the interalveolar septa [1],[2]. The results of this investigation show that *M. bovis*, after initial infection of joint tissue at the inoculation site, subsequently must have spread to other joints most likely by entering the circulation via blood and/or lymphatic vessels. Taking into account the findings of other investigators [15] it seems possible that the agent, after entering the circulation, actively may have invaded peripheral blood mononuclear cells or may have been phagocytosed by such cells and thereafter was transferred to other joints.

The caseonecrotic lesions in the subsynovial connective tissue of the calves histologically closely resemble caseonecrotic foci occurring in lungs of calves after spontaneous or experimental infection [2],[3],[7]. The pathogenetic mechanisms leading to the development of these necrotizing lesions are largely unknown. Findings in lungs of calves with caseonecrotic pneumonia suggest that they mainly occur when other pathogenic bacteria, e.g. *Trueperella pyogenes*, *Pasteurella multocida*, and *Staphylococcus aureus*, are present [7],[16],[17]. In this study, from joints of animals with necrotizing lesions, however, no other bacteria than *M. bovis* were cultured. This strongly indicates that, at least in the joints, the organism itself and/or certain factors or mediators possibly produced by macrophages, neutrophilic granulocytes and other inflammatory cells are able to induce these necrotizing lesions.

Immunohistochemical findings in pneumonic lung tissue of *M. bovis*-infected calves suggest that nitrogen radicals contribute to formation of caseonecrotic pulmonary lesions [6],[8]. Necrotizing lesions in the lungs of *M. bovis*-infected calves are characterized by persistence of *M. bovis* antigens including Vsp antigens and the presence of numerous macrophages expressing iNOS and NT [6]. In general, iNOS is mainly synthesized by activated macrophages following stimulation by different factors, e.g. bacteria, bacterial components such as lipopolysaccharides (LPS), and cytokines, and leads to the production of nitric oxide (NO) from L-arginine. The damaging effects of NO are mediated by peroxynitrite and can be demonstrated by the detection of NT [18]–[20]. As described for the lungs of calves with caseonecrotic pneumonia [6] the necrotic foci in the joints were demarcated by numerous macrophages strongly expressing iNOS and NT indicating production of NO and peroxynitrite.

*In vitro* studies with human and bovine articular cartilage explants have shown that the induction of NO activates matrix metalloproteinases (MMPs) [21]. Possibly, NO produced by macrophages and/or cytokines (IL-1, TNF-α) in inflamed *M. bovis*-infected joints activates MMPs leading to collagen breakdown and destruction of joint tissue. Furthermore, cytokines or MMP’s produced by activated synoviocytes and mononuclear inflammatory cells could be involved in this destructive process.

The results of this study show that, in spite of the developing local immune response of the host, i.e. the demarcation of necrotic foci by numerous phagocytes and other inflammatory cells, *M. bovis* antigens as well as viable organisms are able to persist within caseonecrotic tissue lesions. As in necrotizing lung lesions [6],[7] only single macrophages demarcating the necrotic synovial lesions in calves of this study were positive for MHC class II antigen. This finding suggests that down-regulation of antigen-presenting mechanisms occurs which possibly is caused by marked local production of iNOS and NO by infiltrating macrophages.

The antibodies used in this study for immunolabelling of macrophages detect calprotectin, which is a heterodimer of two calcium-binding proteins. Calprotectin is present in the cytoplasm of neutrophilic granulocytes and reactive tissue macrophages and is secreted extracellularly from stimulated cells or is released as a result of cell disruption or death [22]. Calprotectin is known under several synonyms (complex of S100A8 and S100A9 proteins, macrophage inhibitory factor-related protein MRP8/14, L1L and L1H proteins, calgranulin A/B) and can be induced in human monocytes by certain cytokines (IL-1β, TNF-α) and by LPS. Calprotectin is produced by freshly recruited, classically activated, pro-inflammatory subtypes of macrophages in tissue of different species including cattle [22]–[24] but can also be induced by IL-10 during later stages of inflammation. In this study, low expression of MHC class II by macrophages demarcating the necrotic tissue areas within the joint tissue indicates that these macrophages, possibly under the influence of certain cytokines produced during inflammation, e.g. IL-1 and IFN-γ, are in a stage of diminished activation as discussed for macrophages demarcating necrotic foci in lungs of *M. bovis*-infected calves [7]. Recent investigations in bovine macrophages, however, revealed that the results of the mere expression of calprotectin detected by immunohistochemistry cannot be used to characterize the functionality of macrophages in bovine tissue [25]. Further studies are needed to characterize the phenotypes and the functional characteristics of macrophages involved in necrotic and inflammatory lesions in joint and lung tissue of *M. bovis*-infected calves.

## Conclusions

The results of this study demonstrate that *M. bovis*, following infection of a joint spreads to other joints causing necrotizing and inflammatory changes. The findings that the presence of *M. bovis* antigens in necrotic foci is associated with marked local production of iNOS and NT strongly suggest that in the development of these joint lesions nitritative injury is involved as in necrotizing lung lesions of *M. bovis*- infected calves. Also, the results of this study revealed that, as in pneumonic animals, *M. bovis* is evading the immune response and that the responses both in vaccinated and non-vaccinated calves do not protect them from the development and spread of arthritic lesions. Further investigations are needed to study the interference of *M. bovis* with the specific and innate immune responses of the host.

## Competing interests

The authors declare that they have no competing interests.

## Authors’ contributions

FP designed and carried out the experimental infection, performed the necropsies, and carried out the bacteriological examinations. DLG selected and collected the tissues samples. VRD performed the histopathological and immunohistochemical examination for *M. bovis* antigens, T and B lymphocytes, and MHC class II. KH conducted the immunohistochemistry of iNOS, NT, S100A8, and S100A9. VRD, KH and MH-T performed the analysis and interpretation of the histological and immunohistochemical data. VRD and MH-T drafted the manuscript. All authors revised and finally approved the manuscript.
